# Exploring Deep Learning and Transfer Learning for Colonic Polyp Classification

**DOI:** 10.1155/2016/6584725

**Published:** 2016-10-26

**Authors:** Eduardo Ribeiro, Andreas Uhl, Georg Wimmer, Michael Häfner

**Affiliations:** ^1^Department of Computer Sciences, University of Salzburg, Salzburg, Austria; ^2^Department of Computer Sciences, Federal University of Tocantins, Palmas, TO, Brazil; ^3^St. Elisabeth Hospital, Vienna, Austria

## Abstract

Recently, Deep Learning, especially through Convolutional Neural Networks (CNNs) has been widely used to enable the extraction of highly representative features. This is done among the network layers by filtering, selecting, and using these features in the last fully connected layers for pattern classification. However, CNN training for automated endoscopic image classification still provides a challenge due to the lack of large and publicly available annotated databases. In this work we explore Deep Learning for the automated classification of colonic polyps using different configurations for training CNNs from scratch (or full training) and distinct architectures of pretrained CNNs tested on 8-HD-endoscopic image databases acquired using different modalities. We compare our results with some commonly used features for colonic polyp classification and the good results suggest that features learned by CNNs trained from scratch and the “off-the-shelf” CNNs features can be highly relevant for automated classification of colonic polyps. Moreover, we also show that the combination of classical features and “off-the-shelf” CNNs features can be a good approach to further improve the results.

## 1. Introduction

The leading cause of deaths related to the intestinal tract is the development of cancer cells (polyps) in its many parts. An early detection (when the cancer is still at an early stage) and a regular exam to everyone over an age of 50 years can reduce the risk of mortality among these patients. More specifically, colonic polyps (benign tumors or growths which arise on the inner colon surface) have a high occurrence and are known to be precursors of colon cancer development.

Endoscopy is the most common method for identifying colon polyps and several studies have shown that automatic detection of image regions which may contain polyps within the colon can be used to assist specialists in order to decrease the polyp miss rate [1, 2].

The automatic detection of polyps in a computer-aided diagnosis (CAD) system is usually performed through a statistical analysis based on color, shape, texture, or spatial features applied to the videos frames [3–6]. The main problems for the detection are the different aspects of color, shape, and textures of polyps, being influenced, for example, by the viewing angle, the distance from the capturing camera, or even by the colon insufflation as well as the degree of colon muscular contraction [5].

After detection, the colonic polyps can be classified into three different categories: hyperplasic, adenomatous, and malignant. Kudo et al. [7] proposed the so-called “pit-pattern” scheme to help in diagnosing tumorous lesions once suspicious areas have been detected. In this scheme, the mucosal surface of the colon can be classified into 5 different types designating the size, shape, and distribution of the pit structure [8, 9].

**Figure 1:**
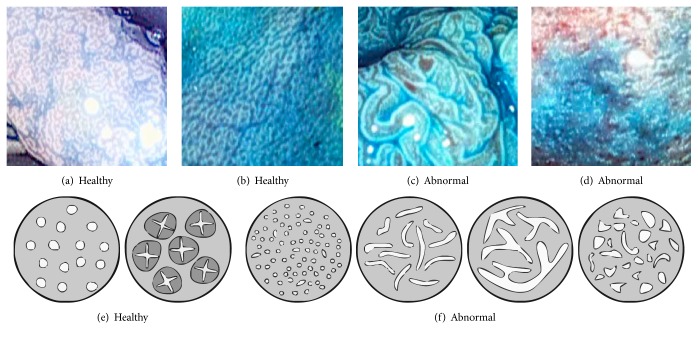
Example images of the two classes (a–d) and the pit-pattern types of these two classes (e–f).

As can be seen in the Figures 1(a)–1(d), these five patterns also allow the division of the lesions into two main classes: (1) normal mucosa or hyperplastic polyps (healthy class) and (2) neoplastic, adenomatous, or carcinomatous structures (abnormal class). This approach is quite relevant in clinical practice as shown in a study by Kato et al. [10].

In the literature, existing computer-aided diagnosis techniques generally make use of feature extraction methods of color, shape, and texture in combination with machine learning classifiers to perform the classification of colon polyps [9, 11, 12]. For example, the dual-tree complex wavelet transform DT-CWT features proved to be quite suitable for the distinction of different types of polyps as can be seen in many works like, for example, [13–15]. Other features were also proved to be quite suitable for colonic polyp classification as the Gabor wavelets [16], vascularization features [17], and directional wavelet transform features [18]. Particularly, in the work of Wimmer et al. [18], using the same 8 colonic polyp databases of this work, an average accuracy of 80.3% was achieved in the best scenario. In this work, we achieve an average accuracy of 93.55% in our best scenario.

The main difficulty of the feature extraction methods is the proper characterization of these patterns due to several factors as the lack or excess of illumination, the blurring due to movement or water injection, and the appearance of polyps [5, 9]. Also, to find a robust and a global feature extractor that summarizes and represents all these pit-pattern structures in a single vector is very difficult and Deep Learning can be a good alternative to surpass these problems. In this work we explore the use of Deep Learning through Convolutional Neural Networks (CNNs) to develop a model for robust feature extraction and efficient colonic polyp classification.

To achieve this, we test the use of CNNs trained from scratch (or full training) and off-the-shelf CNNs (or pretrained) using them as medical imaging feature extractors. In the case of the CNN full training we assume that a feature extractor is formed during the CNN training, adapting to the context of the database and particularly in the case of off-the-shelf CNNs we consider that the patterns learned in the original database can be used in colonoscopy images for colonic polyp classification. In particular, we explore two different architectures for the training from scratch and six different off-the-shelf architectures, describing and analyzing the effects of CNNs in different acquisition modes of colonoscopy images (8 different databases). This study was motivated by recent studies in computer vision addressing the emerging technique of Deep Learning presented in the next section.

## 2. Materials and Methods

### 2.1. Using CNNs on Small Datasets

Some researchers propose replacing handcrafted feature extraction algorithms with Deep Learning approaches that act as features extractor and image classifier at the same time [19]. For example, the Deep Learning approach using CNNs takes advantage of many consecutive convolutional layers followed by pooling layers to reduce the data dimensionality making it, concomitantly, invariant to geometric transformations. Such convolution filters (kernels) are built to act as feature extractors during the training process and recent research indicates that a satisfactorily trained CNN with a large database can perform properly when it is applied to other databases, which can mean that the kernels can turn into a universal feature extractor [19]. Also, Convolutional Neural Networks (CNNs) have been demonstrated to be effective for discriminative pattern recognition in big data and in real-world problems, mainly to learn both the global and local structures of images [20].

Many strategies exploiting CNNs can be used for medical image classification. These strategies can be employed according to the intrinsic characteristics of each database [21] and two of them, mostly used when it comes to CNN training, are described in the following part.

When the available training database is large enough, diverse, and very different from the database used in all the available pretrained CNNs (in a case of transfer learning), the most appropriate approach would be to initialize the CNN weights randomly (training the* CNN trained from scratch*) and train it according to the medical image database for the kernels domain adaptation, that is, to find the best way to extract the features of the data in order to classify the images properly. The main advantage of this approach is that the same method can be used for the extraction of strong features that are invariant to distortion and position at the same time of the image classification. Finally, the Neural Network Classifier can make use of these inputs to delineate more accurate hyperplanes helping the generalization of the network.

This strategy, although ideal, is not widely used due to the lack of large and annotated medical image database publicly available for training the CNN. However, some techniques can assist the CNN training from scratch with small datasets and the most used approach is data augmentation. Basically, in data augmentation, transformations are applied to the image making new versions of it to increase the number of samples in the database. These transformations can be applied in both the training and the testing phase and can use different strategies such as cropping (overlapped or not), rotation, translation, and flipping [22]. Experiments show that using these techniques can be effective to combat overfitting in the CNN training and improve the recognition and classification accuracy [22, 23].

Furthermore, when the database is small, the best alternative is to use an* off-the-shelf CNN* [21]. In this case, using a pretrained CNN, the last or next-to-last linear fully connected layer is removed and the remaining pretrained CNN is used as a feature extractor to generate a feature vector for each input image from a different database. These feature vectors can be used to train a new classifier (such as a support vector machine, SVM) to classify the images correctly. If the original database is similar to the target database, the probability that the high-level features describe the image correctly is high and relevant to this new database. If the target database is not so similar to the original, it can be more appropriate to use higher-level features, that is, features from previous layers of CNN.

In this work, besides using a CNNs trained from scratch, we consider the knowledge transfer between natural images and medical images using off-the-shelf pretrained CNNs. The CNN will project the target database samples into a vector space where the classes are more likely to be separable. This strategy was inspired by the work of Oquab et al. [24], which uses a pretrained CNN on a large database (ImageNet) to classify images in a smaller database (Pascal VOC dataset) with improved results. Unlike that work, rather than copy the weights of the original pretrained CNN to the target CNN with additional layers, we use the pretrained CNN to project data into a new feature space through the propagation of the colonic polyp database into the CNN getting the resultant vector from the last CNNs layer, obtaining a new representation for each input sample. Subsequently, we use the feature vector set to train a linear classifier (e.g., support vector machines) in this representation to evaluate the results as used in [25, 26].

### 2.2. CNNs and Medical Imaging

In recent years there has been an increased interest in machine learning techniques that is based not on hand-engineered feature extractors but using raw data to learn the representations [19].

Among the development of efficient parallel solvers together with GPUS, the use of Deep Learning has been extensively explored in the last years in different fields of application. Deep Learning is intimately related to the use of raw data to do high-level representations of this knowledge through a large volume of annotated data. However, when it comes to the medical area, this type of application is limited by the problem of the lack of large, annotated, and publicly available medical image databases such as the existing natural image databases. Additionally, it is a difficult and costly task to acquire and annotate such images and due to the specific nature of different medical imaging modalities which seems to have different properties according to each modality the situation is even aggravated [21, 27].

Recently, works addressing the use of Deep Learning techniques in medical imaging have been explored in many different ways mainly using CNNs trained from scratch. In biomedical applications, examples include mitosis detection in digital breast cancer histology [28] and neuronal segmentation of membranes in electron microscopy [29]. In Computer-Aided Detection systems (CADe systems), examples include a CADe of pulmonary embolism [30], computer-aided anatomy detection in CT volumes [31], lesion detection in endoscopic images [32], detection of sclerotic spine metastases [33], and automatic detection of polyps in colonoscopy videos [27, 34, 35]. In medical image classification, CNNs are used for histopathological image classification [36], digestive organs classification in wireless capsule endoscopy images [37, 38], and automatic colonic polyp classification [39]. Besides that, CNNs have also been explored to improve the accuracy of CADe systems knee cartilage segmentation using triplanar CNNs [40].

Other recent studies show the potential for knowledge transfer from natural images to the medical imaging domain using off-the-shelf CNNs. Examples include the identification and pathology of X-ray and computer tomography modalities [25], automatic classification of pulmonary perifissural nodules [41], pulmonary nodule detection [26], and mammography mass lesion classification [42]. Moreover, in [26], Van Ginneken et al. show that the combination of CNNs features and classical features for pulmonary nodule detection can improve the performance of the model.

#### 2.2.1. CNNs Trained from Scratch: Architecture

In this section we briefly describe the components of a CNN and how it can be used to perform the CNN from scratch.

**Figure 2:**
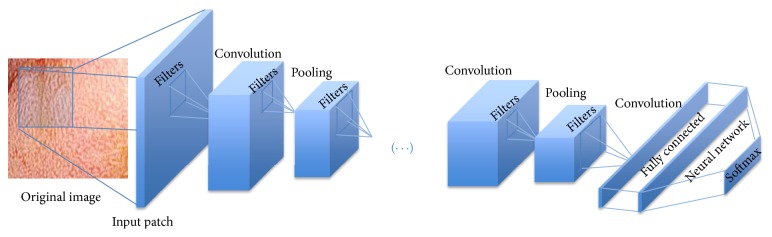
An illustration of the CNN architecture for colonic polyp classification.

A CNN is very similar to traditional Neural Networks in the sense of being constructed by neurons with their respective weights, biases, and activation functions. The structure is basically formed by a sequence of convolution and pooling layers ending in a fully connected Neural Network as shown in Figure 2. Generally, the input of a CNN is *m* × *m* × *d* image (or patch), where *m* × *m* is the dimension of the image and *d* is the number of channels (depth) of the image. The convolutional layer consists of *k* learnable filters (also called kernels) with size *n* × *n* × *d* where *n* ≤ *m* which are convolved with the input image resulting in the so-called activation maps or feature maps. As classic Neural Networks, the convolution layer outputs are submitted to an activation function, for example, the ReLU rectifier function *f*(*x*) = max⁡(0, *x*), where *x* is the neuron input. After the convolution, a pooling layer is included to subsample the image by average functions (mean) or max-pooling over regions of size *p* × *p*. These functions are used to reduce the dimensionality of the data in the following layers (upper layers) and to provide a form of invariance to translation thus making overfitting control. In the convolution and pooling layers the stride has to be specified; the larger the stride, the smaller the overlapping, decreasing the output volume dimensions.

At the end of the CNN there is a fully connected layer as a regular Multilayer Neural Network with the Softmax function that generates a well-formed probability distribution on the outputs. After a supervised training, the CNN is ready to be used as a classifier or as a feature extractor in the case of transfer learning.

#### 2.2.2. CNNs and Transfer Learning

Transfer learning is a technique used to improve the performance of machine learning by harnessing the knowledge obtained by another task. According to Pan and Yang [43], transfer learning can be defined by the following model. We give a domain *D* having two components: a feature space *X* = {*x*_1_, *x*_2_,…, *x*_*n*_} and a probabilistic distribution *P*(*X*); that is, *D* = {*X*, *P*(*X*)}. Also, we give a task *T* with two components: a ground truth *Y* = {*y*_1_, *y*_2_,…, *y*_*n*_} and an objective function *T* = {*Y*, *f*(·)} assuming that this function can be learned through a training database. Function *f*(·) can be used to predict the correspondent class *f*(*x*) of a new instance *x*. From a probabilistic point of view, *f*(*x*) can be written as *P*(*y*∣*x*). In colonic polyp classification, usually, a feature extractor is used to generate the feature space. A given training database *X* associated to the ground truth *Y* consisting of the pairs {*x*_*i*_, *y*_*i*_} is used to train and “learn” the function *f*(·) or *P*(*y*∣*x*) until it reaches a defined and acceptable error rate between the result of the function *f*(*x*) and the ground truth *Y*.

In case of transfer learning, given a source domain *D*_*S*_ = {(*x*_*S*_1__, *y*_*S*_1__), (*x*_*S*_2__, *y*_*S*_2__),…, (*x*_*S*_*n*__, *y*_*S*_*n*__)} and the learning task *T*_*S*_ and the target domain *D*_*T*_ = {(*x*_*T*_1__, *y*_*T*_1__), (*x*_*T*_2__, *y*_*T*_2__),…, (*x*_*T*_*m*__, *y*_*T*_*m*__)} and the learning task *T*_*T*_, transfer learning aims to help improve the learning of the target predictive function *f*_*T*_(·) using the knowledge in *D*_*S*_ and *T*_*S*_, where *D*_*T*_ ≠ *D*_*S*_ and *T*_*T*_ ≠ *T*_*S*_.

Among the various categories of transfer learning, one, called inductive transfer learning, has been used with success in the pattern recognition area. In the inductive transfer learning approach an annotated database is necessary for the source domain as well as for the target domain. In this work, we apply transfer learning between two very different tasks using different labels (*Y*_*T*_ ≠ *Y*_*S*_) and different distributions (*P*(*Y*_*T*_∣*X*_*T*_) ≠ *P*(*Y*_*S*_∣*X*_*S*_)). To bypass the difference between the probability distribution of the images *P*(*X*_*S*_), the last layer from the original function *f*_*S*_(·) directly connected to the classification is removed being replaced by other linear function (as SVM) to adapt it to the new task *T*_*T*_ turning into the function *f*_*T*_(·). In the following sections the functions *f*_*S*_(·) used in this work are presented. Also, the use of transfer learning using pretrained CNNs can help to avoid the problem of lack of data in the medical field. The works of Razavian et al. [19] and Oquab et al. [24] suggest that the use of CNNs intermediate layer outputs can be used as input features to train other classifiers (such as support vector machines) for a number of other applications different from the original CNN obtaining a good performance.

Despite the difference between natural and medical images, some feature descriptors designed especially for natural images are used successfully in medical image detection and classification, for example, texture-based polyp detection [3], Fourier and Wavelet filters for colon classification [18], shape descriptors [44], and local fractal dimension [45] for colonic polyp classification. Additionally, recent studies show the potential of the knowledge transfer between natural and medical images using pretrained (off-the-shelf) CNNs [34, 46].

### 2.3. Experimental Setup

#### 2.3.1. Data

The use of an integrated endoscopic apparatus with high-resolution acquisition devices has been an important object of research in clinical decision support system area. With high-magnification colonoscopies it is possible to acquire images up to 150-fold magnified, revealing the fine surface structure of the mucosa as well as small lesions. Recent work related to classification of colonic polyps used highly-detailed endoscopic images in combination with different technologies divided into three categories: high-definition endoscope (with or without staining the mucosa) combined with the i-Scan technology (1, 2, and 3) [18], high-magnification chromoendoscopy [8], and high-magnification endoscopy combined with narrow band imaging [47].

Specifically, the i-Scan technology (Pentax) used in this work is an image processing technology consisting of the combination of surface enhancement and contrast enhancement aiming to help detect dysplastic areas and to accentuate mucosal surfaces and applying postprocessing to the reflected light being called virtual chromoendoscopy (CVC) [44].

There are three i-Scan modes available: i-Scan1, which includes surface enhancement and contrast enhancement, i-Scan2 that includes surface enhancement, contrast enhancement, and tone enhancement, and i-Scan3 that, besides including surface, contrast, and tone enhancement, increases lighting emphasizing the features of vascular visualization [18]. In this work we use an endoscopic image database (CC-i-Scan Database) with 8 different imaging modalities acquired by an HD endoscope (Pentax HiLINE HD+ 90i Colonoscope) with images of size 256 × 256 extracted from video frames either using the i-Scan technology or without any computer virtual chromoendoscopy (¬CVC).

Table 1 shows the number of images and patients per class in the different i-Scan modes. The mucosa is either stained or not stained. Despite the fact that the frames were originally in high-definition, the image size was chosen (i) to be large enough to describe a polyp and (ii) small enough to cover just one class of mucosa type (only healthy or only abnormal area). The image labels (ground truth) were provided according to their histological diagnosis.

#### 2.3.2. Employed CNN Techniques

Due to the limitation of colonic polyp images to train a good CAD system from scratch, the main elements of the proposed method are defined in order to (1) extract and preprocess images aiming to have a database with a suitable size, (2) use CNNs for learning representative features with good generalization, and (3) enable the use of methods to avoid overfitting in the training phase.

To test the application of a CNN trained from scratch we used the i-Scan1 database without chromoscopy (staining the mucosa) that presents a good performance in the tests using classical features and pretrained CNNs (on average) and subsequently applying the best configuration to the i-Scan3 without chromoscopy database that presented the best results among the classical features results.

In the first experiment of CNN full training, it is proposed that an architecture should be trained with subimages of size 227 × 227 × 3 based on the work of [20] to fit into the chosen architecture. Usually, some simple preprocessing techniques are necessary for the image feature generation. In this experiment we apply normalization by subtracting the mean and dividing by the standard deviation of its elements as in [48] corresponding to local brightness and normalization contrast. We also perform data augmentation by flipping each original image horizontally and vertically and rotating the original image 90° to the right and left. Besides that, we flipped horizontally the rotated images, and then we flipped vertically the horizontally flipped image, totalizing 7 new samples for each original image. After the data augmentation (resulting in 800 images), we randomly extract 75 subimages of size 227 × 227 × 3 from each healthy image and 25 subimages from each abnormal image for the training set to balance the number of images in each class.

Also, in this experiment, to be able to compare the different architectures in a faster way, we used cross-validation evaluation with 10 different CNNs for each architecture. In nine of them, we removed 56 patients for training and used 6 for tests and, in one of them, we removed 54 patients for training and used 8 for test to assure that all the 62 patients are tested. The accuracy result given for each architecture is the average accuracy from each of the 10 CNNs trained based on the final classification of each image between the two classes.

For the second experiment in the CNN full training we propose to extract subimages of size 128 × 128 from the original images using the same approach as in the first experiment. In this case, we explore the hypothesis that the colonic polyp classification with the CNN can be done only with a part of the image, and then we trained the network with smaller subimages instead of the entire image. This helps to reduce the size of the network reducing its complexity and can allow different polyp classifications in the same image using different subimages in different parts of the image. Additionally, choosing smaller regions in a textured image can diminish the degree of intraimage variances in the dataset as the neighborhood is limited.

Besides the different architectures for the training from scratch, we mainly explore six different off-the-shelf CNN architectures trained to perform classification on the ImageNet ILSVRC challenge data. The input of all tested pretrained CNNs has size of 224 × 224 × 3 and the descriptions as well as the details of each CNN are given as follows:

- The* CNN VGG-VD* [49] uses a large number of layers with very small filters (3 × 3) divided into two architectures according to the number of their layers. The CNN* VGG-VD16* has 16 convolution layers and five pooling layers while the CNN* VGG-VD19* has 19 convolution layers, adding one more convolutional layer in three last sequences of convolutional layers. The fully connected layers have 4096 neurons followed by a Softmax classifier with 1000 neurons corresponding to the number of classes in the ILSVRC classification. All the layers are followed by a rectifier linear unit (ReLU) layer to induce the sparsity in the hidden units and reduce the gradient vanishing problem.
- The* CNN-F *(also called Fast CNN) [22] is similar to the CNN used by Alex et al. [20] with 5 convolutional layers. The input image size is 224 × 224 and the fast processing is granted by the stride of 4 pixels in the first convolutional layer. The fully connected layers also have 4096 neurons as the CNN VGG-VD. Besides the original implementation, in this work, we also used the MatConvNet implementation (beta17 [50]) of this architecture trained with batch normalization and minor differences in its default hyperparameters and called here* CNN-F MCN*.
- The* CNN-M* architecture (Medium CNN) [22] also has 5 convolutional layers and 3 pooling layers. The number of filters is higher than the Fast CNN: 96 instead of 64 filters in the first convolution layer with a smaller size. We also use the MatConvNet implementation called* CNN-M MCN*.
- The* CNN-S* (Slow CNN) [22] is related to the “accurate” network from the Overfeat package [51] and also has smaller filters with a stride of 2 pixels in the first convolutional layer. We also use the MatConvNet implementation called* CNN-S MCN*.
- The* AlexNet* CNN [20] has five convolutional layers, three pooling layers (after layers 2 and 5), and two fully connected layers. This architecture is similar to the CNN-F, however, with more filters in the convolutional layers. We also use the MatConvNet implementation called* AlexNet MCN*.
- The* GoogleLeNet* [52] CNN has the deepest and most complex architecture among all the other networks presented here. With two convolutional layers, two pooling layers, and nine modules also called “inception” layers, this network was designed to avoid patch-alignment issues introducing more sparsity in the inception modules. Each module consists of six convolution layers and one pooling layer concatenating these filters of different sizes and dimensions into a single new filter.

In order to form the feature vector using the pretrained CNNs, all images are scaled using bicubic interpolation to the required size for each network, in the case of this work, 224 × 224 × 3. The vectors obtained by the linear layers of the CNN have size of 1024 × 1 for the GoogleLeNet CNN and of 4096 × 1 for the other networks due to their architecture specificities.

#### 2.3.3. Classical Features

To allow the CNN features comparison and evaluation, we compared them with the results obtained by some state-of-the-art feature extraction methods for the classification of colonic polyps [18] shortly explained in the next items.

- *BSAG-LFD*. The Blob Shape adapted Gradient using Local Fractal Dimension method combines BA-LFD features with shape and contrast histograms from the original and gradient image [45].
- *Blob SC*. The Blob Shape and Contrast algorithm [44] is a method that represents the local texture structure of an image by the analyses of the contrast and shape of the segmented blobs.
- *Shearlet-Weibull*. Using the Discrete Shearlet Transform this method adopts regression to investigate dependencies across different subband levels using the Weibull distribution to model the subband coefficient distribution [53].
- *GWT Weibull*. The Gabor Wavelet Transform function can be dilated and rotated to get a dictionary of filters with diverse factors [18] and its frequency using different orientations is used as a feature descriptor also using the Weibull distribution.
- *LCVP*. In the Local Color Vector Patterns approach, a texture operator computes the similarity between neighboring pixels constructing a vector field from an image [12].
- *MB-LBP*. In the Multiscale Block Local Binary Pattern approach [54], the LBP computation is done based on average values of block subregions. This approach is used for a variety image processing applications including endoscopic polyp detection and classification [12].

For the classical features, the classification accuracy is also computed using an SVM classifier, however, with the original images (without resizing) trained using the leave-one-patient-out cross-validation strategy assuring that there are no images from patients of the validation set in the training set as in [55] to make sure the classifier generalizes to unseen patients. This cross-validation is applied to the classical feature extraction methods from the literature as well as to the full training and off-the-shelf CNNs features. The accuracy measure is used to allow an easy comparability of results due to the high number of methods and databases to be compared.

## 3. Results and Discussion

### 3.1. CNNs Trained from Scratch

In the first experiment for the CNN full training, we first use the configuration similar to [20] that can be seen in Table 2 and it can be concluded that the accuracy result was not satisfactory (79%). This can be explained by the fact that Neural Networks involving a large number of inputs require a great amount of computation in training, requiring more data to avoid overfitting (which is not available given the size of our dataset).

For the second experiment, the hyperparameters presented in Table 3 were selected based on the works [48, 56] and empirical adjustment tests in the architecture such as changing the size and number of filters as well as the number of units in the fully connected layer were made and are also shown in Table 3. It can be seen that the architecture CNN-05 obtained the best results, therefore, chosen to perform the subsequent tests.

In the third experiment, with the CNN-05 configuration, we trained one CNN for each patient from the database (leave-one-patient-out (LOPO) cross-validation). Specifically, the results from the CNNs presented in Table 4 are the mean values of the validation set from 62 different CNNs, one for each patient, implemented using the MatConvNet framework [50]. After training the CNN, in the evaluation phase, the final decision for a 256 × 256 pixel image of the dataset is obtained by majority voting of the decisions of all 128 × 128 pixel subimages (patches). One of the advantages of this approach is the opportunity to have a set of decisions available to acquire the final decision for one image. Also, the redundancy of overlapping subimages can increase the system accuracy likewise to give the assurance of certainty for the overall decision.

As it can be seen in Table 4, first we tested with a stride of 1 extracting the maximum number of 128 × 128 subimages available, totalizing 16384 subimages for each image, resulting in an accuracy of 89.00%. This evaluation is very computationally expensive to perform, so we decided to evaluate with different strides resulting in different number of subimages as it is shown in Table 4. We also perform a random patch extraction and it can be concluded that there is not much difference between 16384 subimages or just 25 cropped subimages (accuracy of 91.00%), saving considerable computation time and achieving good results. Besides that, using the same procedure we evaluate the architecture CNN-05 for the i-Scan3 database without staining the mucosa that presented the best results among the classical features and results are presented in Table 5.

For a better comparability of results, we trained an SVM with the extracted vectors from the last fully connected layers (LFCL) and from the prior fully connected layers (PFCL) of CNN-05 as we make in the transfer learning approach explained in the next section. The vectors are extracted from 25 cropped subimages of size 128 × 128 (with stride of 32 pixels) feedforwarded into the CNN-05 subsequently used to train a support vector machine also using the LOPO cross-validation [55]. The results from this approach using the CNN-05 architecture trained with the i-Scan1 and i-Scan3 without staining the mucosa databases are presented in Table 5. As it can be seen, using the last-layer vectors to train an SVM does not improve the results, mainly because the amount of data is not sufficient to generate representative features to be applied into a linear classifier. However, when the CNN is fully trained, the results surpass the classical features results as can be seen also in Table 5 mostly because the last layers are more suitable to design nonlinear hyperplanes in the classification phase. However, the problem of lack of data still is an issue and using all the information in the image would be better than using cropped patches. The significance comparison between the methods will be explored in the next section. Therefore, in order to try solving this problem, we also propose the use of transfer learning by pretrained CNNs that will be also explained in the next section.

### 3.2. Pretrained CNNs

In this section we present the experiments made exploring the 11 different off-the-shelf CNN architectures with the classical features trying to achieve better results than the CNN trained from scratch. As well as in the CNN trained from scratch, we use the i-Scan1 without staining the mucosa database for the first experiments.

In the first experiment, we tested the use of more samples from the same image using overlapping patches by randomly cropping 25 images of size 224 × 224 × 3 of each original image of size 256 × 256 × 3 (resized using bicubic interpolation for the tests presented in Table 8) increasing the database from 100 to 2500 images. The obtained results after the feature extraction performed by the CNN and after the SVM training also using the LOPO cross-validation are presented in Table 6.

It can be observed that, in this case, the use of more samples from the same image does not provide any significant improvement in the results. On the average, resizing the images produces an accuracy of 87.70% while cropping the images produces an average of 84.87%. One of the explanations for this is that, in case of resized images, there is more information about the polyp to provide to the network, so the CNN can abstract more information and form a more robust and intrinsic vector from the actual features of the lesion. However, in three cases (GoogleLeNet, VGG-VD16, and AlexNet MCN), the results using smaller cropped images surpassed the results using the entire image.

In the second experiment, still using i-Scan1 without staining the mucosa database, we also tested the use of other layers of CNNs to extract features.  Table 7 shows the results obtained when the vectors are extracted from the last fully connected layer and when the vectors are from the prior fully connected layer. In the case of the last layer, the results are worse (87.70% against 85.75% on average) because the vectors from the prior fully connected layer are more related to high-level features describing the natural images used for training the original CNNs that are very different from the features to describe colonic polyp images. However, in this case, the results from CNN-F and AlexNet CNN are better using the features from the last fully connected layers.

Based on the results from the two experiments explained before, we tested the methods with all the other databases using the inputs resized to size 224 × 224 × 3 by bicubic interpolation and extracting the features from the prior fully connected layer. The accuracy results for the colonic polyp classification for the 8 different databases are reported in Table 8. As can be seen, the results in Table 8 are divided into three groups: off-the-shelf features, classical features, and the fusion between off-the-shelf features and classical features that will be explained as follows.

Among the 11 pretrained CNNs investigated, the CNNs that present lower performance were GoogleLeNet, CNN-S, and AlexNet MCN. These results may indicate that such networks themselves are not sufficient to be considered off-the-shelf feature extractors for the polyp classification task.

As it can be seen in Table 8, the pretrained CNN that presents the best result on average for the different imaging modalities ($\bar{X}$) is the CNN-M network trained with the MatConvNet parameters (89.74%) followed by the CNN VGG-VD16 (88.59%). These deep models with smaller filters generalize well with other datasets as it is shown in [49], including texture recognition, which can explain the better results in the colonic polyp database. However, there is a high variability in the results and thus it is difficult to draw general conclusions.

Many results obtained from the pretrained CNNs surpassed the classic feature extractors for colonic polyp classification in the literature. The database that presents the best results using off-the-shelf features is the database staining the mucosa without any i-Scan technology (¬CVC, 88.54% on average). In the case of classical features, the database with the best result on average is the database using the i-Scan3 technology without staining the mucosa (81.61%).

To investigate the differences in the results we assess the significance of them using the McNemar test [57]. By means of this test we analyze if the images from a database are classified differently or similarly when comparing two methods. With a high accuracy it is supposed that the methods will have a very similar response, so the significance level *α* must be small enough to differentiate between classifying an image as correct or incorrect.

**Figure 3:**
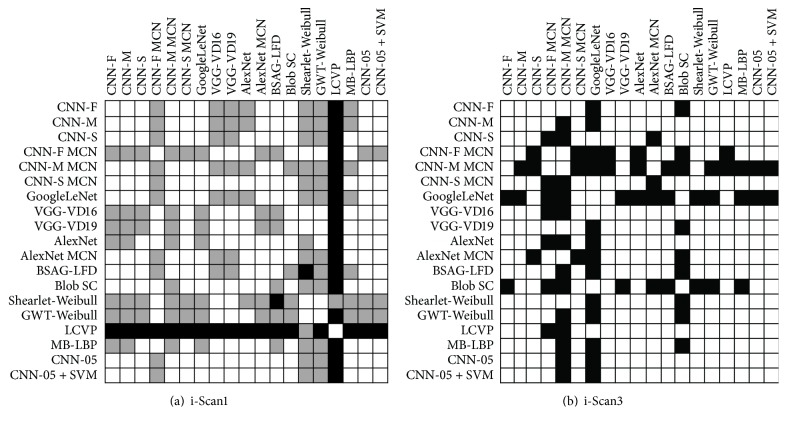
Results of the McNemar test for the i-Scan1 (a) and i-Scan3 (b) databases without staining. A black square in the matrix means that the methods are significantly different with significance level *α* = 0.01 and a grey square in (a) means that the methods are significantly different with significance level *α* = 0.05. If the square is white then there is no significant difference between the methods.

The test is carried out on the databases i-Scan3 and i-Scan1 without staining the mucosa using significance level *α* = 0.01 with all the off-the-shelf CNNS, all the classical features, and the CNN-05 architecture trained from scratch. The results are presented in Figure 3. It can be observed by the black squares (indicating significantly differences) that, among the pretrained CNNs, in the i-Scan1 database the results are not significantly different and in the i-Scan3 database the CNN-M MCN and GoogleLeNet present the most significantly different results comparing to the other CNNs. It also can be seen that the CNN-05 does not have significantly different results comparing to the other CNNs in the i-Scan1 database and has significantly different results with CNN-M MCN and GoogleLeNet in the i-Scan3 database.

Also, in Figure 3, when comparing the classical feature extraction methods with the CNNs features it can be seen that there is a quite different response among the results in i-Scan3 database, especially for CNN-M MCN that is significantly different from all the classical methods with the exception of the Shearlet-Weibull method. The CNN-05 and CNN-05 + SVM did not present significantly different results with the classical features (except with LCVP in i-Scan1 database) and with the pretrained CNNs (except with CNN-M and GoogleLeNet in i-Scan3 database). Likewise, the methods with high accuracy in the i-Scan3 database (BSAG-LFD, VGG-VD16, and VGG-VD19) are not found to be significantly different.

In the i-Scan1 database, with the significance level *α* = 0.05, the results are not significantly different in general (except for LCVP features). However, with the significance level *α* = 0.01, the significance results represented by the grey squares in Figure 3(a) show that the two databases presented different correlation between methods which means that it is difficult to predict a good feature extractor that can satisfy both databases at the same time.

Observing the methods that presented significantly different results in Figure 3 and with good results in Table 8 we decided to produce a feature level fusion in the feature vectors concatenating them to see if the features can complement each other. It can be seen in Figure 3 that the two most successful CNNs CNN-M MCN and VGG-VD16 are significantly different from each other in both databases and the feature level fusion of these two vectors improve the results from 89.74% and 88.59%, respectively, to an accuracy of 90.58% in average as can be seen in Table 8 (Fusion 5/8).

In Figure 3(b) it can also be observed that the results from CNN-M MCN are significantly different to the classical features BSAG-LFD in the i-Scan3 database. With the feature level fusion of these two features the accuracy increases to 91.03% on average. Concatenating the three feature vectors (CNN-M MCN, VGG-VD16, and BSAG-LFD) leads to an even better accuracy: 93.22%. It is interesting to note that in both databases the results from CNN-M MCN and VGG-VD16 are significantly different. Besides that, BSAG-LFD results are significantly different to VGG-VD16 in database i-Scan1. Furthermore, BSAG-LFD results are significantly different to CNN-M MCN in database i-Scan3 which can explain the improvement in the feature level fusion between these three methods.

Making the fusion with these two off-the-shelf CNNs (CNN-M MCN and VGG-VD16) to other classical feature vectors also increases the accuracy as it can be seen in Table 8 (Fusion 5/8/14 and Fusion 5/8/15).

When we add to the vector Fusion 5/8/12 one more classical feature (MB-LBP) that is also significantly different to CNN-M MCN in database i-Scan3 and at the same time significantly different to BSAG-LFD in database i-Scan1, the result outperforms all the previous approaches: 93.55% as it can be seen in Table 8.

**Figure 4:**
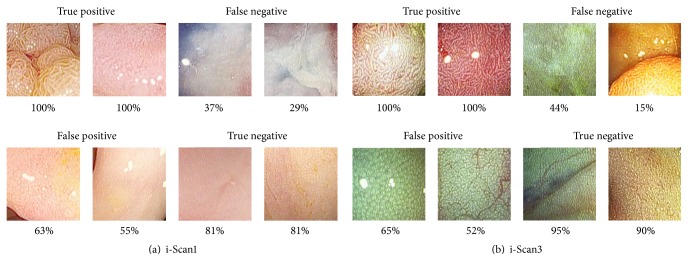
Example results of the classification in agreement from the methods tested in the McNemar test for each prediction outcome.

In Figure 4 we present some example images from the classification results of all the methods used in the McNemar test with the higher agreement for each prediction outcome. The percentage above each image shows the average classification rate of the prediction. For example, in the i-Scan1 database and i-Scan3 database (Figures 4(a) and 4(b)), the two images presented in the true positive box were classified as such in all classifiers. However, from i-Scan3 database, in the case of the false negative box, one image had 44% of misclassification and another 15% of misclassification in average.

Comparing the results from all off-the-shelf CNNs and classical features with the CNN-05 trained from scratch using the databases i-Scan1 and i-Scan3 in Table 8 it can be observed that the full training CNN outperformed the results obtained by the classical features and some of the pretrained CNNs. This approach can be considered an option for automatic colonic polyp classification, although the training time and processing complexity are not worthwhile if comparing to the off-the-shelf features.

## 4. Conclusion

In this work, we propose to explore Deep Learning and Transfer Learning approach using Convolutional Neural Networks (CNNs) to improve the accuracy of colonic polyp classification based on the fact that databases containing large amounts of annotated data are often limited for this type of research. For the training of CNNs from scratch, we explore data augmentation with image patches to increase the size of the training database and consequently the information to perform the Deep Learning. Different architectures were tested to evaluate the impact of the size and number of filters in the classification as well as the number of output units in the fully connected layer.

We also explored and evaluated several different pretrained CNNs architectures to extract features from colonoscopy images by knowledge transfer between natural and medical images providing what is called off-the-shelf CNNs features. We show that the off-the shelf features may be well suited for the automatic classification of colon polyps even with a limited amount of data.

Besides the fact that the pretrained CNNs were trained with natural images, the 4096 features extracted from CNN-M MCN and VGG-16 provided a good feature descriptor of colonic polyps. Some reasons for the success of the classification include the training with a large range of different images providing a powerful extractor joining the intrinsic features from the images such as color, texture, and shape in the same architecture reducing and abstracting these features in just one vector. Also, the combination of classical features with off-the-shelf features yields the best prediction results complementing each other. It can be concluded that Deep Learning using Convolutional Neural Networks is a good option for colonic polyp classification and the use of pretraining CNNs is the best choice to achieve the best results being improved by feature level fusion with classical features. In future work we plan to use this strategy to also test the detection of colonic polyps directly into video frames and evaluate the performance in real time applications as well as to use this strategy in other endoscopic databases such as automatic classification of celiac disease.

## Figures and Tables

**Table 1:** Number of images and patients per class of the CC-i-Scan databases gathered with and without CC (staining) and computed virtual chromoendoscopy (CVC).

i-Scan mode	No staining				Staining			
	¬CVC	i-Scan1	i-Scan2	i-Scan3	¬CVC	i-Scan1	i-Scan2	i-Scan3
*Non-neoplastic*								
Number of images	39	25	20	31	42	53	32	31
Number of patients	21	18	15	15	26	31	23	19
*Neoplastic*								
Number of images	73	75	69	71	68	73	62	54
Number of patients	55	56	55	55	52	55	52	47
Total number of images	112	100	89	102	110	126	94	85

**Table 2:** CNN configuration for input subimages of size 227 × 227 × 3 and its respective accuracy in %.

Size of inputs	Number of convolutional filters/size								Connected layer
	Layer 1	Layer 2	Layer 3	Layer 4	Layer 5	Layer 6	Layer 7	Layer 8	
227 × 227 × 3	96/11 × 11	256/5 × 5	384/3 × 3	384/3 × 3	256/3 × 3	384/3 × 3	384/3 × 3	4096/6 × 6	4096

Accuracy: 79.00									

**Table 3:** Accuracy results from different CNN configurations for inputs of size 128 × 128 × 3 in %.

Network index	Number of convolutional filters/size			Connected layer	Acc
	Layer 1	Layer 2	Layer 3		
CNN-01	48/7 × 7	72/4 × 4	512/5 × 5	512	76.00
CNN-02	48/11 × 11	72/5 × 5	512/6 × 6	512	84.00
CNN-03	24/11 × 11	48/5 × 5	1024/6 × 6	1024	86.00
CNN-04	24/11 × 11	72/4 × 4	2048/5 × 5	2048	80.00
CNN-05	48/11 × 11	72/5 × 5	1024/6 × 6	1024	*87.00*

**Table 4:** Accuracy of different strides for overlapping subimages in the CNN-05 evaluation for i-Scan1 database in %.

Stride	Number of subimages	Accuracy
1	16384	89.00
5	676	89.00
20	49	90.00
32	25	*91.00*
48	9	87.00
Random	9	87.00
Random	25	89.00
Random	49	89.00

**Table 5:** Accuracy of CNN-05 architecture comparing to classical features for the i-Scan1 and i-Scan3 databases in %.

Methods	i-Scan1	i-Scan3
CNN-05	*91.00*	*89.00*
CNN-05 + SVM − LFCL	83.00	72.55
CNN-05 + SVM − PFCL	80.00	66.67
BSAG-LFD	86.87	82.87
Blob SC	83.33	75.22
Shearlet-Weibull	76.67	86.80
GWT-Weibull	78.67	84.28
LCVP	66.00	77.12
MB-LBP	80.67	83.37

**Table 6:** Results from i-Scan1 database with images resized to 224 × 224 and cropped in 25 patches of size 224 × 224.

	CNN-F	CNN-M	CNN-S	CNN-F MCN	CNN-M MCN	CNN-S MCN	Google LeNet	VGG VD16	VGG VD19	AlexNet	AlexNet MCN	$\overset{-}{X}$
Resizing image	89.33	90.67	90.00	82.00	90.67	91.42	90.67	85.33	82.67	87.33	84.67	**87.70**
Cropping 25 images	84.00	82.67	84.67	78.67	84.67	88.67	91.29	89.67	78.67	85.33	85.33	84.87

**Table 7:** Results from i-Scan1 database with images resized to 224 × 224 using the last fully connected layer and the prior fully connected layer.

	CNN-F	CNN-M	CNN-S	CNN-F MCN	CNN-M MCN	CNN-S MCN	Google LeNet	VGG VD16	VGG VD19	AlexNet	AlexNet MCN	$\overset{-}{X}$
Prior fully connected layer	89.33	90.67	90.00	82.00	90.67	91.42	90.67	85.33	82.67	87.33	84.67	**87.70**
Last fully connected layer	90.67	84.67	85.33	78.67	88.00	89.33	90.67	84.67	79.33	81.33	90.67	85.75

**Table 8:** Accuracies of the methods for the CC-i-Scan databases in %.

Methods	No staining				Staining				
	¬CVC	i-Scan1	i-Scan2	i-Scan3	¬CVC	i-Scan1	i-Scan2	i-Scan3	$\overset{-}{X}$
1: CNN-F	86.16	89.33	80.65	88.41	86.52	81.40	84.22	80.62	84.66
2: CNN-M	87.45	90.67	81.38	83.58	87.99	89.55	87.40	90.53	87.31
3: CNN-S	88.03	90.00	87.01	77.33	87.25	82.68	87.40	75.54	84.41
4: CNN-F MCN	88.84	82.00	73.15	90.73	85.78	89.55	89.72	83.15	85.36
5: CNN-M MCN	89.53	90.67	*88.88*	*94.66*	86.97	89.29	87.40	90.53	**89.74**
6: CNN-S MCN	90.12	*91.42*	81.38	79.85	89.18	*93.49*	81.10	84.77	86.41
7: GoogleLeNet	79.65	90.67	72.43	74.51	88.27	80.46	75.60	84.08	80.70
8: VGG-VD16	87.45	85.33	86.38	79.65	*92.47*	89.80	*95.26*	*92.38*	88.59
9: VGG-VD19	83.49	82.67	83.88	87.71	*92.47*	83.98	94.46	85.59	86.78
10: AlexNet	*91.40*	87.33	75.65	89.32	87.71	83.03	84.22	79.24	84.73
11: AlexNet MCN	89.42	84.67	78.88	83.78	89.36	83.55	81.10	78.32	83.63
$\overset{-}{X}$	87.41	87.70	80.88	84.50	88.54	86.07	86.17	84.06	85.67

12: BSAG-LFD	*86.27*	*86.87*	*84.60*	82.87	70.20	*80.63*	78.78	71.39	**80.20**
13: Blob SC	77.67	83.33	82.10	75.22	59.28	78.83	66.13	59.83	72.79
14: Shearlet-Weibull	73.72	76.67	79.60	*86.80*	*81.30*	69.91	72.38	*83.63*	78.00
15: GWT-Weibull	79.75	78.67	70.25	84.28	*81.30*	74.54	77.17	83.39	78.66
16: LCVP	76.60	66.00	47.75	77.12	77.45	79.00	70.01	69.56	70.43
17: MB-LBP	78.26	80.67	81.38	83.37	69.29	70.60	77.22	78.32	77.38
$\overset{-}{X}$	78.71	78.70	74.28	81.61	73.13	75.58	73.61	74.35	76.24

Fusion 5/8	88.84	85.33	83.88	92.14	93.12	90.49	96.88	94.00	90.58
Fusion 5/12	92.79	*92.67*	88.88	*96.98*	87.71	90.49	88.26	90.53	91.03
Fusion 5/8/12	*95.94*	90.00	88.88	92.14	92.30	91.43	97.63	*97.46*	93.22
Fusion 5/8/14	91.51	88.67	87.10	93.75	*94.68*	91.43	*98.44*	95.85	92.67
Fusion 5/8/15	90.91	90.00	88.88	92.14	93.94	89.80	96.88	95.61	92.27
Fusion 5/8/12/14	93.38	88.00	*91.38*	93.75	93.49	*92.12*	97.63	94.92	93.08
Fusion 5/8/12/17	93.38	90.00	*91.38*	93.75	92.75	*92.12*	97.63	*97.46*	**93.55**

CNN-05	—	91.00	—	89.00	—	—	—	—	—
CNN-05 + SVM	—	83.00	—	72.55	—	—	—	—	—
